# Comparison of the prognosis among different age groups in elderly patients with hip fracture

**DOI:** 10.4103/0019-5413.38577

**Published:** 2008

**Authors:** Tetsuo Hagino, Satoshi Ochiai, Masanori Wako, Eiichi Sato, Shingo Maekawa, Yoshiki Hamada

**Affiliations:** Department of Orthopaedic Surgery, National Hospital Organization, Kofu National Hospital, Yamanashi, Japan; 1 Interdisciplinary Graduate School of Medicine and Engineering, University of Yamanashi, Yamanashi, Japan

**Keywords:** Elderly population, hip fracture, prognosis

## Abstract

**Background:**

The outcome of treatment of hip fractures in different age groups in the elderly population is largely unknown. Hence, we stratified elderly patients with hip fracture into age groups and compared the prognosis in various age groups.

**Materials and Methods:**

Among 459 patients with hip fracture treated at our hospital from 1997, 430 patients aged 65 years or above at the time of injury were studied. The patients comprised 98 males and 332 females and the ages at injury ranged from 65 to 103 years (mean 83.4 years). There were 167 cases of femoral neck fracture and 263 cases of trochanteric fractures. Surgery was performed in 383 cases, while 47 cases were treated conservatively. The subjects were classified by age into young-old for those aged 65-74 years (group A, n = 55), middle-old for those aged 75-84 years (group B, n = 172), old-old for those aged 85-94 (group C, n = 180), and oldest-old for those aged 95 years or above (group D, n = 23). The functional and survival prognosis at discharge in each group was investigated.

**Results:**

Numbers of patients who were ambulatory at discharge among those ambulatory before injury were 43 of 49 (87.8%) in group A, 113 of 152 (74.3%) in group B, 86 of 138 (62.3%) in group C, and 5 of 14 (35.7%) in group D, showing worse recovery of walking ability as age advanced. Among those ambulatory before injury, 42 patients in group A, 139 patients in group B, 130 patients in group C, and 12 patients in group D underwent surgery and of these patients, 38 patients (90.5%) in group A, 109 patients (78.4%) in group B, 83 patients (63.8%) in group C, and 5 patients (41.7%) in group D were ambulatory at discharge. On the other hand, the numbers of patients who were ambulatory at discharge among those receiving conservative treatment were 5 of 7 (71.4%) in group A, 4 of 13 (30.8%) in group B, 3 of 8 (37.5%) in group C, and 0 of 2 (0%) in group D, showing better walking ability in surgical patients than in conservatively treated patients even in the elderly. There were two in-hospital deaths in group B, 11 in group C, and two in group D. Five of the 15 deaths were inoperable cases due to poor performance status at admission.

**Conclusion:**

Walking ability at discharge and survival prognosis worsened as age advanced. On the other hand, since surgical cases achieved better walking ability than conservatively treated cases, efforts should be made to achieve better functional prognosis even in the old-olds, including surgery together with early ambulation and rehabilitation.

## INTRODUCTION

The elderly population in Japan reached another record high both in number and proportion according to the latest statistics of September 15, 2007; the number of elderly aged 65 years or above was 27.44 million, occupying 21.5% of the total population.^1^ The proportion of the elderly population continues to rise and is expected to reach 30% in 2025. Accompanying this trend, the number of hip fractures also increases and the number of elderly patients with impaired walking ability or becoming bed-ridden after treatment is anticipated to increase. Therefore, in conducting treatment for hip fractures, it is important to know the patient characteristics and the prognosis in different age groups of the elderly population. However, our search of the literature found no report that compares hip fracture patients by age group. In this study, we stratified elderly patients with hip fracture into age groups and compared the characteristics, treatment methods, and the outcome of various age groups.

## MATERIALS AND METHODS

Among 459 patients with hip fracture treated at our hospital from 1997, 430 patients aged 65 years or above at the time of injury were included in this study. There were 98 males and 332 females, with ages at injury ranging from 65 to 103 years (mean 83.4 years). One hundred sixty-seven cases were femoral neck fractures and 263 cases were trochanteric fractures. Femoral neck fractures were classified by Garden classification and trochanteric fractures by Evans classification. Furthermore, femoral neck fractures were divided into impacted and nondisplaced neck (Garden stages I and II, n = 71) and displaced neck (Garden stages III and IV, n = 96); and trochanteric fractures into stable trochanteric (Evans type 1, groups 1 and 2, n = 163) and unstable trochanteric (Evans type 1, groups 3 and 4; and type 2, n = 100). The surgical method of first choice was osteosynthesis for impacted and nondisplaced neck fractures, hemiarthroplasty for displaced neck fractures, and osteosynthesis for trochanteric fractures. Surgery was performed in 383 cases; comprising open reduction and internal fixation using sliding hip screw (n=165) gamma nail (n=39) Twin Hook system^2^ (n=34) Hansson pin (n=50) cannulated cancellous hip screw (n=19) Ender nail (n=2), and femoral head arthroplasty (n=74).

Postoperative rehabilitation varied during the study period. Before 2000, a program of beginning partial weight-bearing from 2 weeks after surgery was conducted. Since 2000, critical pathway has been adopted and subsequently modified. Recently, the patient rests for 2 days after surgery. On the third day, wheelchair usage is commenced and muscle strengthening and joint range of motion training are started at the rehabilitation room. From day 7 after surgery, walking training with partial weight-bearing is started. The remaining 47 cases received conservative treatments because the patient or family declined surgery, or the patient had severe dementia or systemic comorbidities. Our rehabilitation program given as conservative treatment for femoral neck fracture is as follows. For impacted and nondisplaced neck, after the pain is relieved by bed rest for 3-5 days after injury, wheelchair usage is commenced and standing and walking training is started at the rehabilitation room. Weight-bearing is started 5 weeks after injury. For displaced neck of femur, union is not expected and after several days of indirect traction, wheelchair usage is commenced. Standing and walking training is conducted as long as the pain can be tolerated. For trochanteric hip fractures, after 4 weeks of traction therapy in bed, wheelchair usage is commenced by the fifth week, and standing and walking training is conducted. In all patients, upper and lower muscle training is started in bed immediately after admission. The goal set for discharge was recovery to the same level of walking ability as before injury, but in the case that walking ability had reached a plateau, the ability at discharge was taken as the goal. The subjects were classified by age at injury into young-old for those aged 65-74 years (group A, n = 55), middle-old for those aged 75-84 years (group B, n = 172), old-old for those aged 85-94 (group C, n = 180), and oldest-old for those aged 95 years or above (group D, n = 23). The performance status at admission as well as functional and survival outcome at discharge were investigated and compared among four groups. Statistical analysis was performed using the Statcel software (OMS Publishing Inc., Tokyo, Japan). Mann-Whitney's *U*-test, one-factor ANOVA, and Mantel-Haenszel procedure were used for comparisons between groups. A *P*-value less than 0.05 was considered to indicate a significant difference.

## RESULTS

First, the characteristics of the 430 patients at admission were investigated. The proportion of males was 34.5% in group A compared with 13% in group D, showing that the frequency of males decreased as age advanced in elderly patients with hip fracture. The number of cases who were ambulatory before injury was 49 of 55 cases (89.1%) in group A, 152 of 172 cases (88.4%) in group B, 138 of 180 cases (76.7%) in group C, and 14 of 23 cases (60.9%) in group D, indicating deteriorated walking ability as age increased. While dementia, anemia, and electrocardiographic abnormality were more common as the subjects became older, fracture types were not significantly different among age groups [Table 1].

**Table 1:** Characteristics of 430 hip fracture patients

	Group A 65-74 yrs (*n* = 55) No. (%)	Group B 75-84 yrs (*n* = 172) No. (%)	Group C 85-94 yrs (*n* = 180) No. (%)	Group D >95 yrs (*n* = 23) No. (%)	*P*-value*
Male	19 (34.5)	42 (24.4)	34 (18.9)	3 (13.0)	0.0097
Ambulatory prior to fracture	49 (89.1)	152 (88.4)	138 (76.7)	14 (60.9)	<0.0005
Femoral neck fracture	27 (49.1)	69 (40.1)	65 (36.1)	6 (26.1)	0.80
Dementia	6 (10.9)	70 (40.7)	92 (51.1)	12 (52.2)	<0.00001
Anemia	16 (29.1)	74 (43.0)	101 (56.1)	14 (60.9)	<0.0001
Electrolyte abnormality	12 (21.8)	45 (26.2)	52 (28.9)	9 (39.1)	0.14
Abnormal lung function	1 (1.8)	19 (11.0)	21 (11.7)	4 (17.4)	0.063
Abnormal ECG (electrocardiogram)	10 (18.2)	66 (38.4)	75 (41.7)	16 (69.6)	<0.0005
Chronic systemic diseases	25 (45.5)	111 (64.5)	113 (62.8)	9 (39.1)	0.70

* Mann-Whitney's *U*-test

The proportions of patients who received operative treatment in the four groups ranged from 83.6 to 90.1%, with no significant differences among groups. Duration between admission and discharge tended to be shorter in group D, but the difference was not significant. No death occurred during hospitalization in group A, but there were two deaths (1.2%) in group B, 11 deaths (6.1%) in group C, and two deaths (8.7%) in group D, showing an increased in-hospital mortality rate as age advanced [Table 2]. Five of the 15 deaths were among inoperable cases because of poor general condition at admission.

**Table 2:** In-hospital data of 430 hip fracture patients

	Group A 65-74 yrs (*n* = 55)	Group B 75-84 yrs (*n* = 172)	Group C 85-94 yrs (*n* = 180)	Group D >95 yrs (*n* = 23)	*P*-value
Operative treatment [number of patients (%)]	46 (83.6%)	155 (90.1%)	162 (90.0%)	20 (87.0%)	0.50*
Duration between admission and discharge (days, Mean ± SD)	75.3 ± 43.2	63.9 ± 34.2	65.4 ± 44.3	48.9 ± 41.0	0.063**
In-hospital death [number of patients (%)]	0 (0%)	2 (1.2%)	11 (6.1%)	2 (8.7%)	0.0016*

* Mann-Whitney's *U*-test;

** One-factor ANOVA

Comparing the places of residence at the time of fracture and discharge, the proportion of patients returning to their own home after discharge decreased as age increased, with only 57 cases (33.7%) in group C and five cases (23.8%) in group D returning to their own home [Table 3].

**Table 3:** Place of residence at the time of fracture and discharge

Place of residence	Admission* [number of patients (%)]				Discharge destination** [number of patients (%)]			
		
	Group A 65-74 yrs (*n* = 55)	Group B 75-64 yrs (*n* = 172)	Group C 85-94 yrs (*n* = 180)	Group D >95 yrs (*n* = 23)	Group A 65-74 yrs (*n* = 55)	Group B 75-84 yrs (*n* = 170)	Group C 85-94 yrs (*n* = 169)	Group D >95 years (*n* = 21)
Own home	48 (87.3)	120 (69.8)	110 (61.1)	9 (39.1)	38 (69.1)	76 (44.7)	57 (33.7)	5 (23.8)
Nursing home or Hospital	7 (12.7)	52 (30.2)	70 (38.9)	14 (60.9)	17 (30.9)	94 (55.3)	112 (66.3)	16 (76.2)

Values exclude those who died during hospitalization,

* *P* < 0.00005;

** *P* < 0.00001; Mann-Whitney's *U*-test

The numbers of ambulatory patients at discharge among those who were ambulatory before injury were 43 of 49 (87.8%) in group A, 113 of 152 (74.3%) in group B, 86 of 138 (62.3%) in group C, and 5 of 14 (35.7%) in group D, showing poorer recovery of walking ability as age advanced [Table 4]. Among those ambulatory before injury, 42 patients in group A, 139 patients in group B, 130 patients in group C, and 12 patients in group D underwent surgery; and of these patients, 38 patients (90.5%) in group A, 109 patients (78.4%) in group B, 83 patients (63.8%) in group C, and five patients (41.7%) in group D were ambulatory at discharge. On the other hand, the numbers of patients who were ambulatory at discharge among those receiving conservative treatment were 5 of 7 (71.4%) in group A, 4 of 13 (30.8%) in group B, 3 of 8 (37.5%) in group C, and 0 of 2 (0%) in group D. The above results showed better recovery of walking ability in surgically treated patients than in conservatively treated patients even in the elderly [Table 5], implying that treatment method is related to walking ability at discharge.

**Table 4:** Ambulation prognosis at discharge among those ambulatory before injury

Ambulation prognosis	Group A 65-74 yrs (*n* = 49) No. (%)	Group B 75-84 yrs (*n* = 152) No. (%)	Group C 85-94 yrs (*n* = 138) No. (%)	Group D >95 yrs (*n* = 14) No. (%)
Ambulatory	43 (87.8)	113 (74.3)	86 (62.3)	5 (35.7)
Nonambulatory	6 (12.2)	39 (25.7)	52 (37.7)	9 (64.3)

*P* < 0.00005; Mann-Whitney's *U*-test

**Table 5:** Relation between treatment modality and ambulation status at the time of discharge from hospital

Ambulation prognosis	Operative treatment [number of patients (%)]				Nonoperative treatment [number of patients (%)]			
		
	Group A 65-74 years (*n* = 42)	Group B 75-84 years (*n* = 139)	Group C 85-94 years (*n* = 130)	Group D >95 years (*n* = 12)	Group A 65-74 years (*n* = 7)	Group B 75-84 years (*n* = 13)	Group C 85-94 years (*n* = 8)	Group D >95 years (*n* = 2)
Ambulatory	38 (90.5)	109 (78.4)	83 (63.8)	5 (41.7)	5 (71.4)	4 (30.8)	3 (37.5)	0 (0)
Nonambulatory	4 (9.5)	30 (21.6)	47 (36.2)	7 (58.3)	2 (28.6)	9 (69.2)	5 (62.5)	2 (100)

Values exclude those who were not able to walk independently prior to fracture, *P* < 0.00005; Mantel-Haenszel procedure

## DISCUSSION

The proportion of hip fracture patients who are ambulatory at discharge has been reported to range from 60 to 80%.^3^ The factors influencing walking ability after treatment include age, pre-injury walking ability, status of dementia, and status of chronic systemic disease.^4^,^6^ Holt *et al*.^7^ investigated the walking ability of 50 patients aged 95 years and older with hip fracture and reported that while 96% of the patients were ambulatory at the time of fracture, only 36% regained ambulation at the final follow-up at a mean 29.3 months. In the present study, results of the subject characteristics before injury showed poorer walking ability and higher prevalence of dementia as age advanced. The proportions of ambulatory patients before injury who regained walking ability at discharge were 87.8% in group A, 74.3% in group B, and 62.3% in group C, which were similar to previous reports and showed poor recovery of walking ability as age advanced and the rate was markedly lower at 35.7% in group D patients aged 95 years or older.

Regarding survival outcome, the in-hospital mortality rate of elderly patients with proximal femoral fracture has been reported to range from 5 to 8%.^3^ Hasegawa *et al*.^8^ identified the risk factors associated with mortality following hip fracture to be male sex, older age, high American Academy of Anesthesiology (ASA) grade, dementia, and residence in an institution. In our study, the in-hospital mortality rates of 1.2% in group B, 6.1% in group C, and 8.7% in group D also demonstrated increased deaths during hospitalization as age advanced.

Thorngren *et al*.^9^ reported that age was a highly discriminating factor after a hip fracture in the elderly, with a pronounced influence on the rehabilitation pattern. Arinzon *et al*.^10^ compared the young elderly aged 65-74 years and the old-old elderly aged 85 years and older with hip fractures. Their study showed that the old-old elderly patients were more functional-dependent before fracture, had more comorbid diseases and had malnutrition as shown by low hemoglobin and serum albumin levels, and their functional outcome was poor. We have reported that the status of anemia and dementia at admission is closely related to functional outcome.^11^ In the present study also, older elderly patients with hip fracture had lower walking ability before injury, were more likely to live in institutions, and had dementia and anemia more commonly. Furthermore, as age advanced, fewer patients were able to return to their own home after discharge and their walking ability as well as survival outcome worsened, and this trend was particularly marked in those aged 95 years or older. Holt *et al*.^7^ reported that patients over 95 years were unlikely to recover their independence and 96% required permanent institutional care. Thorngren *et al*.^9^ reported that fewer patients returned to their former place of living after discharge as age advanced, indicating that age had a critical effect on planning rehabilitation pattern after hip fracture in the elderly. We have previously reported poor outcome in patients with hip fracture who underwent conservative treatments.^12^ In the present study also, patients who underwent operative treatment regained walking ability better than those who underwent conservative treatments. The findings of this study thus indicate that active surgery, early ambulation, and early rehabilitation should be conducted even in the old-old elderly patients to achieve better functional outcome.

No benefits in any form have been received or will be received from a commercial party related directly or indirectly to the subject of this article.
